# Environmental Drivers of Metabolic Plasticity in *Rubia cordifolia* From the Western Himalaya: Implications for Phytochemical Diversity and Antioxidant Potential

**DOI:** 10.1002/cbdv.71747

**Published:** 2026-09-30

**Authors:** Deepti Tiwari, Sandeep Rawat, Veena Pandey, Vinod C. Joshi, Indra D. Bhatt, R. C. Sundriyal

**Affiliations:** ^1^ Centre For Biodiversity Conservation and Management G. B. Pant National Institute of Himalayan Environment, Kosi‐Katarmal Almora Uttarakhand India; ^2^ G. B. Pant National Institute of Himalayan Environment Sikkim Regional Centre Gangtok Sikkim India; ^3^ Division of Environment Science Indian Agriculture Research Institute New Delhi India; ^4^ Wildlife Institute of India, Chandrabani Dehradun Uttarakhand India; ^5^ Department of Forestry and Natural Resources HNB Garhwal University Srinagar, Garhwal Uttarakhand India; ^6^ Sparsh Himalaya University Dehradun Uttarakhand India

**Keywords:** active ingredients, altitudinal variation, habitat variability, metabolic plasticity, secondary metabolites

## Abstract

*Rubia cordifolia* L. is an important Himalayan medicinal plant valued for its diverse therapeutic properties. The present study investigated the influence of altitude, habitat conditions, and edaphic factors on nutritional composition, phytochemicals, and antioxidant activity of *R. cordifolia* populations collected from different habitats (open grassy, shady, and moist shady) in the western Himalaya. Significant variations (*p* ≤ 0.05) were observed in edaphic factors, nutritional components and phytochemicals, antinutritional factors and antioxidant activities. High‐altitude populations showed higher accumulation of total phenolics (52.59 mg GAE/g dw), flavonoids (120.84 mg QE/g dw), alizarin (2.68 mg/g dw), and mollugin (23.77 mg/g dw). Correlation analysis revealed a significant positive correlation of altitude with phenolics, flavonoids, alizarin, catechin, amino acids, and thiamine. Whereas, soil pH demonstrated negative correlations with several metabolites. In contrast, altitude was negatively correlated (*p* ≤ 0.01) with salicylic acid in leaves and stems and tannin content in roots. Moist shady habitats showed greater accumulation of nutritional and bioactive compounds compared with open grassy and shady habitats. The results suggest that altitude, habitat conditions, and soil factors collectively regulate metabolic plasticity and antioxidant potential in *R. cordifolia*. High‐altitude populations occuring in moist shady habitat may presents valuable germplasm for conservation, cultivation, and pharmaceutical applications.

## Graphical abstract

Environmental heterogeneity drives remarkable metabolic plasticity in *Rubia cordifolia* with significant variation in nutritional constituents, phytochemicals, bioactive compounds, and antioxidant activity among natural populations. High‐altitude populations exhibited higher accumulation of phenolics, flavonoids, alizarin, mollugin, and antioxidant activity, while moist shady habitats favored higher nutritional and phytochemical accumulation. Soil moisture and nutrients such as organic carbon, nitrogen, potassium, and pH showed significant associations with metabolite profiles and antioxidant potential.

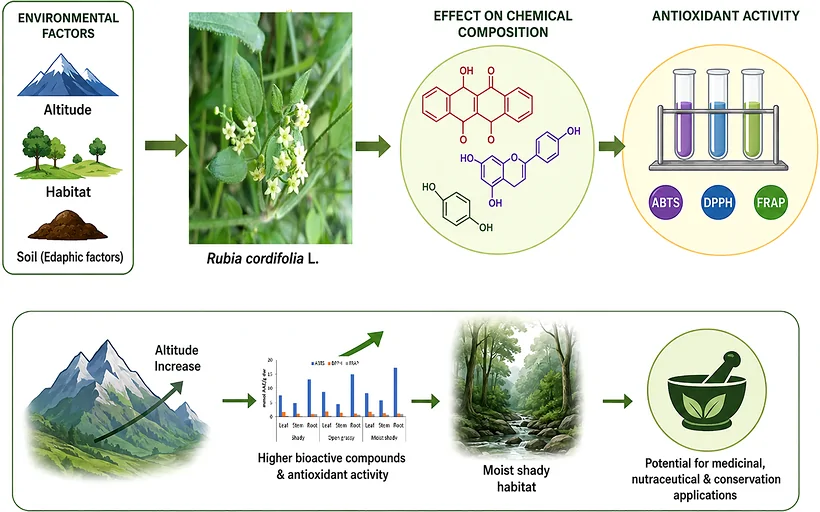

## 1 Introduction

The importance of medicinal plants as a fundamental component of global healthcare systems has gained considerable attention in recent decades. The therapeutic efficacy of medicinal plants is largely attributed to their bioactive metabolites such as polyphenols (phenolic acids, flavonoids, anthocyanins, stilbenes, and lignans), terpenoids, and glycosides [1]. The accumulation of these bioactive constituents is strongly influenced by various environmental factors, such as temperature, soil characteristics, water and nutrient availability, carbon dioxide concentrations, salinity, pollutants, radiation (light, ultraviolet and ionizing radiation), and ozone [1, 2]. Among these factors, altitude plays a significant role by altering ecological conditions (soil nutrients, temperature, and water availability), which directly or indirectly influence plant physiology, biological processes, and secondary metabolite biosynthesis [2, 3]. Previous studies have demonstrated that increasing altitude enhances the accumulation of secondary metabolites such as curcumin in turmeric and gingerol in *Zingiber* species [4]. In contrast, the berberine content in *Berberis asiatica* has been reported to decrease with increasing altitude [5]. These observations indicate that plant‐environment interactions significantly influence the biosynthesis of secondary metabolites. The phenomenon of phenotypic plasticity enables plants to modulate their metabolic pathways in response to diverse biotic and abiotic stresses [1, 6].

*Rubia cordifolia* L. (commonly known as Indian madder or Manjistha) belonging to the family Rubiaceae, is an important medicinal plant. It is widely distributed across the Indian subcontinent, Eastern and Southeast Asia, and tropical Africa [7]. *R. cordifolia* is found throughout the lower hills of the Indian Himalayan Region, as well as in the Western and Eastern Ghats and the northeastern states of India, within an altitudinal range of 800–2800 m above sea level [8, 9]. In traditional Indian systems of medicine, particularly Ayurveda, *R. cordifolia* is highly valued for its blood‐purifying properties and diverse therapeutic applications [10]. The indigenous people of Uttarakhand Himalaya use the roots of *R. cordifolia* for the treatment of dysentery, jaundice, hepatic disorders, ulcers, stomach pain, snake bites, and a variety of skin and blood diseases [11, 12, 13]. Decoctions prepared from the leaves and stems are traditionally used as vermifuges [13]. A gel formulated from an anthraquinone‐rich fraction of *R. cordifolia* roots was reported to have potent antiacne activity [14]. Several studies have reported the presence of diverse nutritional and bioactive constituents in *R. cordifolia* with significant biological activities [15, 16, 17]. For instance, alizarin isolated from *R. cordifolia* contains a wide range of biological activities [15, 16, 17]. For example, alizarin derived from *R. cordifolia* roots exhibited a modulatory role against the genotoxicity of mutagens, namely 4‐nitro‐o‐phenylenediamine (NPD) and 2‐aminofluorene (2‐AF) [18]. Rubiadin, a major anthraquinone isolated from an ethanol extract of *R. cordifolia* roots, possesses antioxidant and hepatoprotective activity [19].

Although several studies have investigated the traditional uses, metabolite composition, and pharmacological properties of *R. cordifolia* [9, 20], comprehensive information on the collective roles of altitude, habitat conditions, and edaphic factors on its nutritional quality, phytochemical composition, bioactive compounds, and antioxidant potential remains limited. Environmental heterogeneity, particularly variations in altitude, habitat type, and soil properties, significantly influences metabolic plasticity in medicinal plants, resulting in distinct patterns of metabolite accumulation and antioxidant activity among natural populations. Understanding the effects and relative contributions of these environmental influences is essential for identifying elite natural populations and germplasm resources with superior medicinal quality and for developing evidence‐based conservation and cultivation strategies. Therefore, the present study was undertaken to (i) quantify key metabolites, including phenolic and other bioactive compounds together with antioxidant activities across different populations, (ii) evaluate the effects of altitude, habitat type, and soil characteristics on the nutritional and phytochemical composition of *R. cordifolia* populations, and (iii) determine the relationships between environmental variables and metabolite accumulation. The findings of this study may provide a scientific basis for identifying suitable habitats and populations with enhanced medicinal potential, thereby supporting targeted harvesting, cultivation, conservation, and commercial utilization strategies for *R. cordifolia*.

## 2 Results

### 2.1 Edaphic Factors

The physicochemical analysis of the soil collected from the nine study sites revealed significant spatial heterogeneity (*p* ≤ 0.05), indicating that the *R. cordifolia* populations occur under edaphically diverse conditions (Table 1). The soil moisture content was higher in the RC‐9 population (17.97 %) and lowest in the RC‐1 population (14.28 %). In contrast, the highest pH value was recorded in the RC‐1 population (7.35) and the lowest in the RC‐5 population (6.13). The organic carbon ranged from 4.03 (RC‐6) to 5.46 % (RC‐7) and total nitrogen ranged from 0.32% (RC‐1 & RC‐2) to 0.52% (RC‐7). Total phosphorus levels were generally low, with the highest (0.11%) found in the RC‐6 population. Total potassium content peaked in the RC‐4 population (0.31%).

**TABLE 1:** Site characteristics and physicochemical characteristics of soil of targeted populations of *Rubia cordifolia*.

Location/ District	Site Code	Latitude	Longitude	Altitude (m asl)	Forest type	Habitat	Moisture (%)	pH	Organic carbon (%)	Total nitrogen (%)	Total phosphorus (%)	Total potassium (%)
Kosi	RC‐1	29°39'10''	79°47'11''	1300	Pine	Shady	14.28 ± 0.37^a^	7.35 ± 0.03^ef^	4.16 ± 0.34^a^	0.32 ± 0.04^a^	0.06 ± 0.02^abc^	0.26 ± 0.00^cd^
Dwarahat	RC‐2	29°46'35''	79°26'17''	1407	Pine	Shady	14.61 ± 0.36^ab^	7.07 ± 0.01^def^	4.63 ± 0.55^ab^	0.32 ± 0.10^a^	0.10 ± 0.00^bc^	0.30 ± 0.00^f^
Semalkote	RC‐3	29°39'21''	80°02'44''	1500	Deodar Conifer	Open grassy	14.72 ± 0.46^ab^	7.06 ± 0.03^def^	4.75 ± 0.50^ab^	0.38 ± 0.09^a^	0.09 ± 0.00^abc^	0.28 ± 0.00^dc^
Lobanj	RC‐4	29°52'22''	79°35'14''	1575	Pine	Shady	15.79 ± 0.42^abc^	6.95 ± 0.37^cde^	4.36 ± 0.25^ab^	0.39 ± 0.08^a^	0.07 ± 0.01^abc^	0.31 ± 0.00^f^
Berinag	RC‐5	29°46'13''	80°03'11''	1726	Pine	Open grassy	15.88 ± 0.46^abc^	6.13±0.04^a^	4.10 ± 0.29^a^	0.39±0.08^a^	0.10 ± 0.00^bc^	0.25±0.00^bc^
Nag	RC‐6	29°41'23''	80°02'59''	2000	Banj‐Oak	Moist shady	15.51 ± 0.29^ab^	6.80 ± 0.03^cd^	4.03 ± 0.30^a^	0.46±0.16^a^	0.11 ± 0.00^c^	0.29 ± 0.01^ef^
Tallital‐ Nainital	RC‐7	29°23'25''	79°27'29''	2035	Banj‐Oak	Shady	15.57 ± 0.42^ab^	6.38 ± 0.03^f^	5.46 ± 0.32^b^	0.52±0.05^a^	0.04 ± 0.00^a^	0.26 ± 0.00^c^
Mallital‐Nainital	RC‐8	29°22'59''	79°27'54''	2100	Surai Conifer	Moist shady	16.08 ± 1.12^bc^	6.26 ± 0.04^ab^	5.20 ± 0.28^ab^	0.50±0.10^a^	0.09 ± 0.03^abc^	0.27 ± 0.00^cd^
Dunagiri	RC‐9	29°48'53''	79°26'17''	2500	Banj‐Oak	Moist shady	17.97 ± 0.41^d^	6.63 ± 0.06^bc^	5.46 ± 0.21^ab^	0.50±0.05^a^	0.05 ± 0.00^ab^	0.24 ± 0.00^ab^

Values of soil parameters are presented as mean value of three replicates followed by ± standard error. Different letters in a column indicate significant variation (*p* ≤ 0.05) according to Duncan's multiple‐range test (DMRT). Type of forest as Pine: *Pinus roxburghii*, Deodar Conifer: *Cedrus deodara*, Banj‐Oak: *Quercus leucotrichophora*, Surai Conifer: *Cupressus torulosa*.

### 2.2 Nutritional Content

The nutritional profile of *R. cordifolia* also varied significantly (*p* ≤ 0.05) among the different populations, demonstrating that environmental conditions exert a strong influence on primary metabolism (Table 2). Higher total carbohydrate (leaf: 24.31 mg/g; stem: 11.0 mg/g; root: 3.31 mg/g dw) and total protein (leaf: 2.32 mg/g; stem: 1.11 mg/g; root: 0.99 mg/g dw) contents were recorded in the RC‐9 population. Total free amino acids also peaked in RC‐9 for leaves (2.12 mg/g) and roots (3.15 mg/g), while in stems, the highest value was recorded in RC‐8 (1.85 mg/g). Similarly, higher ascorbic acid content in the leaves (5.67 mg/g dw) and roots (4.831 mg/g dw) was recorded in the RC‐9 population, while in the stems, significantly higher ascorbic acid (5.79 mg/g dw) was recorded in the RC‐8 population. Thiamine content in the leaves (1.73 mg/g dw) was significantly higher in the populations of RC‐6 and RC‐9, while in stems (1.47 mg/g dw) and roots (1.84 mg/g dw) higher thiamine content was demonstrated in RC‐8 and RC‐9 populations, respectively. Factorial analysis revealed that specific nutritive constituents varied significantly (*p* ≤ 0.05) among plant parts and populations, either individually or in combination (Table S1).

**TABLE 2:** Nutritional components in the different populations of *R. cordifolia*.

Population code	Total carbohydrates (mg/g)			Total protein (mg/g)			Total free amino acids (mg/g)			Total ascorbic acid (mg/g)			Total thiamine (mg/g)		
	Leaf	Stem	Root	Leaf	Stem	Root	Leaf	Stem	Root	Leaf	Stem	Root	Leaf	Stem	Root
RC‐1	21.40 ± 0.00^a^	10.71 ± 0.01^a^	2.81 ± 0.00^d^	1.41 ± 0.01^a^	0.83 ± 0.01^b^	0.70 ± 0.01^c^	1.38 ± 0.02^a^	1.26 ± 0.01^a^	2.86 ± 0.01^b^	5.57 ± 0.02^bc^	5.50 ± 0.02^b^	4.67 ± 0.02^bc^	1.59 ± 0.01^b^	1.39 ± 0.01^c^	1.76 ± 0.01^bc^
RC‐2	22.0 ± 0.01^e^	10.40 ± 0.01^e^	3.15 ± 0.00^f^	1.43 ± 0.01^a^	0.80 ± 0.00^a^	0.65 ± 0.00^b^	1.43 ± 0.01^a^	1.46 ± 0.01^b^	2.94 ± 0.02^c^	5.58 ± 0.02^c^	5.47 ± 0.01^b^	4.71 ± 0.02^bc^	1.69 ± 0.02^f^	1.41 ± 0.01^d^	1.79 ± 0.01^c^
RC‐3	21.61 ± 0.01^b^	10.63 ± 0.00^b^	2.65 ± 0.01^c^	1.55 ± 0.00^c^	0.89 ± 0.01^c^	0.064 ± 0.00^b^	1.76 ± 0.01^b^	1.55 ± 0.03^c^	2.86 ± 0.01^b^	5.66 ± 0.02^d^	5.73 ± 0.02^de^	4.56 ± 0.02^a^	1.64 ± 0.00^c^	1.33 ± 0.00^a^	1.75 ± 0.00^c^
RC‐4	21.63 ± 0.02^b^	10.82 ± 0.01^b^	2.42 ± 0.02^a^	1.49 ± 0.01^b^	0.85 ± 0.02^b^	0.58 ± 0.01^a^	1.72 ± 0.01^b^	1.43 ± 0.01^b^	2.79 ± 0.02^a^	5.61 ± 0.02^cd^	5.63 ± 0.02^c^	4.74 ± 0.01^cd^	1.54 ± 0.01^a^	1.31 ± 0.02^a^	1.77 ± 0.01^bc^
RC‐5	21.52 ± 0.00^ab^	10.49 ± 0.02^ab^	2.63 ± 0.00^b^	1.71 ± 0.01^d^	0.89 ± 0.02^c^	0.75 ± 0.01^d^	1.83 ± 0.01^c^	1.58 ± 0.01^c^	2.83 ± 0.01^ab^	5.48 ± 0.02^a^	5.25 ± 0.02^a^	4.58 ± 0.03^a^	1.66 ± 0.01^de^	1.33 ± 0.01^a^	1.79 ± 0.01^c^
RC‐6	22.15 ±0.00^e^	10.60 ±0.01^e^	2.85 ± 0.00^e^	1.85 ± 0.01^f^	0.88 ± 0.02^c^	0.71 ± 0.01^c^	1.85 ± 0.01^c^	1.60 ± 0.01^c^	3.12 ± 0.01^e^	5.62 ± 0.02^cd^	5.71 ± 0.02^d^	4.63 ± 0.05^ab^	1.73 ± 0.01^g^	1.42 ± 0.01^d^	1.77 ± 0.01^bc^
RC‐7	21.70 ± 0.01^c^	10.62 ± 0.01^c^	2.62 ± 0.01^b^	1.79 ± 0.00^e^	0.96 ± 0.01^d^	0.91 ± 0.00^e^	1.96 ± 0.02^d^	1.76 ± 0.02^d^	3.02 ± 0.02^d^	5.51 ± 0.02^bc^	5.62 ± 0.00^c^	4.67 ± 0.02^bc^	1.65 ± 0.02^cd^	1.36 ± 0.01^b^	1.78 ± 0.01^c^
RC‐8	21.90 ± 0.02^d^	10.78 ± 0.00^d^	2.89 ± 0.01^e^	1.93 ± 0.00^g^	1.07 ± 0.00^e^	0.97 ± 0.01^f^	1.93 ± 0.02^d^	1.85 ± 0.01^e^	3.01 ± 0.02^d^	5.66 ± 0.02^d^	5.79 ± 0.01^e^	4.80 ± 0.02^de^	1.67 ± 0.01^e^	1.47 ± 0.01^f^	1.78 ± 0.01^c^
RC‐9	24.31 ± 0.02^f^	11.00 ± 0.02^f^	3.31 ± 0.01^g^	2.32 ± 0.02^h^	1.11 ± 0.01^f^	0.99 ± 0.01^f^	2.12 ± 0.01^e^	1.80 ± 0.01^de^	3.15 ± 0.02^e^	5.67 ± 0.02^d^	5.78 ± 0.02^e^	4.83 ± 0.01^e^	1.73 ± 0.01^g^	1.45 ± 0.01^e^	1.84 ± 0.01^d^
Average	22.02 ± 0.01	10.67 ± 0.01	2.81 ± 0.01	1.72 ± 0.01	0.92 ± 0.01	0.70 ± 0.01	1.77 ± 0.01	1.58 ± 0.03	2.95 ± 0.02	5.59 ± 0.02	5.61 ± 0.02	4.68 ± 0.01	1.65 ± 0.01	1.38 ± 0.01	1.78 ± 0.01

Values are presented as mean value of three replicates followed by ± standard error. Different letters in a column indicate significant variation (*p* ≤ 0.05) according to Duncan's multiple‐range test (DMRT).

### 2.3 Antinutritional Content

The assessment of antinutritional factors revealed significant (*p* ≤ 0.05) variations that could impact the nutritional utility of the plant (Table 3). Phytic acid, known for its ability to chelate minerals and reduce their bioavailability, was found to be significantly higher in the leaves (1.60 mg/g) and roots (1.94 mg/g) of the RC‐9 population. The highest phytic acid content in stems (1.38 mg/g) was recorded in the RC‐5 population. The highest trypsin inhibitory activity in leaves (3.36 mg/g), stems (2.02 mg/g), and roots (1.14 mg/g) was observed in RC‐5, RC‐4, and RC‐6 populations, respectively.

**TABLE 3:** Antinutritional components in the different populations of *R. cordifolia*.

Population code	Phytic acid (mg/g)			Trypsin inhibitory activity (mg/g)		
	Leaf	Stem	Root	Leaf	Stem	Root
RC‐1	1.13 ± 0.01^a^	1.02 ± 0.02^bc^	1.58 ± 0.03^a^	1.94 ± 0.14^a^	1.66 ± 0.04^a^	0.83 ± 0.04^c^
RC‐2	1.41 ± 0.02^b^	1.13 ± 0.04^c^	1.73 ± 0.02^b^	2.11 ± 0.05^a^	1.97 ± 0.02^bc^	0.41 ± 0.04^a^
RC‐3	1.09 ± 0.03^a^	0.82 ± 0.03^a^	1.49 ± 0.05^a^	3.28 ± 0.07^d^	1.91 ± 0.12^abc^	1.08 ± 0.09^de^
RC‐4	1.40 ± 0.02^b^	1.03 ± 0.03^bc^	1.73 ± 0.03^b^	1.94 ± 0.02^a^	2.02 ± 0.12^c^	0.61 ± 0.09^b^
RC‐5	1.37 ± 0.02^b^	1.38 ± 0.05^d^	1.54 ± 0.04^b^	3.36 ± 0.03^d^	1.91 ± 0.09^abc^	0.89 ± 0.07^cd^
RC‐6	1.15 ± 0.02^a^	0.97 ± 0.03^b^	1.52 ± 0.04^a^	2.55 ± 0.02^b^	1.75 ± 0.04^ab^	1.14 ± 0.07^e^
RC‐7	1.42 ± 0.08^b^	1.06 ± 0.01^bc^	1.60 ± 0.02^ab^	2.08 ± 0.04^a^	1.72 ± 0.05^ab^	0.75 ± 0.04^bc^
RC‐8	1.05 ± 0.02^a^	1.02 ± 0.04^bc^	1.71 ± 0.04^a^	3.00 ± 0.08^c^	1.72 ± 0.07^ab^	1.05 ± 0.02^de^
RC‐9	1.60 ± 0.04^c^	1.00 ± 0.03^bc^	1.94 ± 0.02^c^	2.05 ± 0.05^a^	1.86 ± 0.07 ^abc^	0.94 ± 0.02^cde^
Average	1.29 ± 0.03	1.05 ± 0.02	1.65 ± 0.02	2.48 ± 0.10	1.83 ± 0.03	0.85 ± 0.04

Values are presented as mean value of three replicates followed by ± standard error. Different letters in a column indicate significant variation (*p* ≤ 0.05) according to Duncan's multiple‐range test (DMRT).

### 2.4 Total Phenolics, Flavonoids, and Tannin Contents

Secondary metabolites, particularly phenolics, displayed strong population‐dependent variation (*p* ≤ 0.05), strongly supporting the hypothesis of environmentally induced chemical plasticity (Table 4). Higher total phenolic content in leaves (52.59 mg GAE/g dw) and stems (16.34 mg GAE/g dw) was recorded in the RC‐9 population, while in roots, the highest total phenolic content (19.38 mg GAE/g dw) was observed in the RC‐8 population. Similarly, total flavonoids (leaf: 120.84 mg QE/g, stem: 45.90 ± 0.35 mg QE/g) were higher in the RC‐9 population, whereas in roots, the highest total flavonoids (68.17 mg QE/g dw) were found in the RC‐8 population. The total tannin content in leaves (25.57 mg TAE/g dw), stems (13.26 mg TAE/g dw), and roots (15.58 mg TAE/g dw) was significantly (*p* ≤ 0.05) higher in RC‐9, RC‐2, and RC‐1 populations, respectively.

**TABLE 4:** Total phenolics, flavonoids, and tannins in the different populations of *R. cordifolia*.

Population code	Total Phenolics (mg GAE/g)			Total Flavonoids (mg QE/g)			Total Tannins (mg TAE/g)		
	Leaf	Stem	Root	Leaf	Stem	Root	Leaf	Stem	Root
RC‐1	21.57 ± 0.18^b^	5.41 ± 0.1^a^	16.07 ± 0.05^b^	53.50 ± 0.35^a^	26.30 ± 0.13^a^	33.37 ± 0.35^a^	11.16 ± 0.06^a^	11.88 ± 0.18^d^	15.58 ± 0.09^g^
RC‐2	22.79 ± 0.34^c^	5.25 ± 0.0^a^	15.81 ± 0.16^ab^	52.97 ± 0.53^a^	27.50 ± 0.35^abc^	34.04 ± 0.35^ab^	12.05 ± 0.06^b^	13.26 ± 0.02^e^	14.88 ± 0.09^e^
RC‐3	22.79 ± 0.29^c^	7.27 ± 0.1^c^	17.89 ± 0.18^d^	52.04 ± 0.46^a^	27.90 ± 0.35^bcd^	38.97 ± 0.35^d^	11.22 ± 0.06	9.16 ± 0.05^c^	15.05 ± 0.01^f^
RC‐4	17.19 ± 0.18^a^	5.14 ± 0.0^a^	15.33 ± 0.24^a^	52.17 ± 0.48^a^	27.90 ± 0.58^bcd^	34.97 ± 0.35^b^	11.96 ±0.02^b^	5.08 ±0.01^ab^	11.09 ±0.01^a^
RC‐5	25.03 ±0.56^d^	8.55 ±0.1^d^	19.27 ±0.28^fg^	56.30 ±0.81^b^	28.57 ±0.13^cde^	33.64 ± 0.23^ab^	14.15 ± 0.02^c^	5.81 ± 0.02^b^	13.40 ± 0.04^d^
RC‐6	32.55 ± 0.14^f^	8.55 ± 0.3^f^	18.74 ± 0.23^ef^	52.30 ± 0.66^a^	29.64 ± 0.46^e^	37.50 ± 0.48^c^	15.17 ± 0.24^d^	9.74 ± 1.24^c^	13.26 ± 0.02^c^
RC‐7	29.41 ± 0.09^e^	8.45 ± 0.1^e^	16.93 ± 0.16^c^	84.57 ± 0.35^c^	27.10 ± 0.53^ab^	42.04 ± 0.23^e^	16.72 ± 0.07^e^	4.42 ± 0.02^a^	13.42 ± 0.00^d^
RC‐8	34.10 ± 0.26^g^	7.17 ± 0.1^g^	19.38 ± 0.14^g^	88.30 ± 0.35^d^	29.10 ± 0.48^d^	68.17 ± 0.35^f^	18.02 ± 0.04^f^	8.70 ± 0.41^c^	13.14 ± 0.02^c^
RC‐9	52.59 ± 0.09^h^	16.34 ± 0.1^h^	18.37 ± 0.24^de^	120.84 ± 1.00^e^	45.90 ± 0.35^f^	67.10 ± 0.87^f^	25.57 ± 0.05^g^	9.00 ± 0.04^c^	12.46 ± 0.25^b^
Average	28.63 ± 1.92	8.07 ± 0.63	17.53 ± 0.05	68.11 ± 4.53	29.99 ± 1.12	43.31 ± 2.60	15.11 ± 0.87	8.56 ± 0.56	13.59 ± 0.25

Values are presented as mean value of three replicates followed by ± standard error. Different letters in a column indicate significant variation (p ≤ 0.05) according to Duncan's multiple‐range test (DMRT).

### 2.5 Polyphenolic and Bioactive Compounds

HPLC analysis provided a detailed profile of individual phenolic acids (gallic acid, catechin, and salicylic acid), revealing specific responses to environmental gradients, which varied significantly (*p* ≤ 0.05) among the populations (Table 5). Gallic acid in the leaves (2.10 mg/g dw) and stems (1.95 mg/g dw) was significantly (*p* ≤ 0.05) higher in the RC‐8 population, while the RC‐9 population exhibited the highest gallic acid in the root (2.01 mg/g dw). Catechin levels were relatively lower but showed significant (*p* ≤ 0.05) variation. Leaves of the RC‐7 population contained the highest catechin (0.28 mg/g). Stems peaked in the RC‐3 population (0.26 mg/g), while roots contained the highest levels in the RC‐4 population (0.07 mg/g). Interestingly, salicylic acid (SA), an important signaling molecule, displayed a negative association with altitude, particularly in leaves. It was significantly (*p* ≤ 0.05) higher in the leaves of the low‐altitude RC‐1 population (1.67 mg/g) and was notably not detected in the leaves of the RC‐8 population. In roots, it was only detected in RC‐1 (0.22 mg/g) and RC‐9 (0.25 mg/g) populations. Factorial analysis revealed that specific phenolic compounds varied significantly (*p* ≤ 0.05) among plant parts and populations, either individually or in combination (Table S1).

**TABLE 5:** Polyphenolic and bioactive compounds in the different populations of *R. cordifolia*.

Population Code	Gallic acid (mg/g)			Catechin (mg/g)			Salicylic acid (mg/g)			Alizarin (mg/g)			Purpurin (mg/g)			Mollugin (mg/g)
	Leaf	Stem	Root	Leaf	Stem	Root	Leaf	Stem	Root	Leaf	Stem	Root	Leaf	Stem	Root	Root
RC‐1	1.88 ± 0.02^d^	1.66 ± 0.01^a^	1.81 ± 0.02^c^	0.03 ± 0.01^b^	0.06 ± 0.01^e^	0.02 ± 0.00^ab^	1.67 ± 0.02^d^	0.22 ± 0.03^a^	0.22 ± 0.02^a^	0.67 ± 0.01^b^	1.45 ± 0.01^a^	0.31 ± 0.01^a^	1.14 ± 0.01^c^	1.18 ± 0.01^bc^	1.36 ± 0.02^a^	6.77 ± 0.02^c^
RC‐2	1.78 ± 0.03^c^	1.86 ± 0.01^d^	1.85 ± 0.02^e^	0.08 ± 0.01^e^	0.07 ± 0.01^d^	0.02 ± 0.00^ab^	0.93 ± 0.01^c^	0.20 ± 0.02^b^	ND	0.64 ± 0.00^a^	0.66 ± 0.02^a^	0.31 ± 0.01^a^	1.12 ± 0.00^b^	0.66 ± 0.01^a^	1.66 ± 0.01^d^	ND
RC‐3	2.05 ± 0.02^f^	1.91 ± 0.02^f^	1.62 ± 0.01^a^	0.06 ± 0.02^d^	0.26 ± 0.03^a^	0.03 ± 0.01^ab^	0.29 ± 0.01^a^	ND	ND	0.82 ± 0.01^c^	0.53 ± 0.00^b^	0.50 ± 0.02^b^	1.30 ± 0.00^h^	1.17 ± 0.01^bc^	3.68 ± 0.01^h^	ND
RC‐4	1.59 ± 0.02^a^	1.80 ± 0.01^c^	1.83 ± 0.03^d^	0.01 ± 0.00^a^	0.02 ± 0.00^a^	0.07 ± 0.01^c^	0.25 ± 0.01^a^	ND	ND	1.34 ± 0.02^e^	1.36 ± 0.01^e^	2.16 ± 0.00^e^	1.22 ± 0.01^f^	1.36 ± 0.02^cd^	3.16 ± 0.01^g^	2.44 ± 0.03^a^
RC‐5	1.60 ± 0.04^a^	1.74 ± 0.03^b^	1.78 ± 0.01^b^	0.01 ± 0.00^a^	0.08 ± 0.00^d^	0.02 ± 0.01^ab^	0.40 ± 0.01^a^	ND	ND	1.71 ± 0.01^h^	1.68 ± 0.02^f^	2.48 ± 0.01^f^	1.10 ± 0.01^b^	1.19 ± 0.02^bc^	2.84 ± 0.01^f^	2.73 ± 0.01^b^
RC‐6	1.67 ± 0.01^b^	1.79 ± 0.02^c^	1.95 ± 0.02^f^	0.05 ± 0.00^c^	0.04 ± 0.01^b^	0.01 ± 0.00^b^	0.35 ± 0.02^a^	ND	ND	1.46 ± 0.01^f^	1.13 ± 0.00^d^	1.21 ± 0.03^d^	1.16 ± 0.00^d^	1.43 ± 0.16^cd^	1.95 ± 0.00^e^	ND
RC‐7	1.99 ± 0.01^e^	1.87 ± 0.02^e^	1.82 ± 0.01^cd^	0.28 ± 0.02^g^	0.04 ± 0.01^b^	0.01 ± 0.00^a^	0.26 ± 0.02^a^	ND	ND	1.21 ± 0.00^d^	1.30 ± 0.01^f^	2.50 ± 0.01^f^	1.25 ± 0.01^g^	1.20 ± 0.02^bc^	1.63 ± 0.01^c^	19.84 ± 0.02^d^
RC‐8	2.10 ± 0.04^f^	1.95 ± 0.04^h^	1.97 ± 0.01^g^	0.16 ± 0.01^f^	0.04 ± 0.01^b^	0.05 ± 0.02^b^	ND	ND	ND	1.57 ± 0.02^g^	1.55 ± 0.00^c^	0.88 ± 0.01^b^	1.19 ± 0.02^e^	1.17 ± 0.01^bc^	1.60 ± 0.00^c^	ND
RC‐9	1.89 ± 0.01^d^	1.93 ± 0.01^f^	2.01 ± 0.01^h^	0.07 ± 0.00^e^	0.09 ± 0.01^e^	0.03 ± 0.00^b^	0.62 ± 0.10^b^	ND	0.25 ± 0.02^b^	1.73 ± 0.02^i^	1.68 ± 0.02^g^	2.68 ± 0.01^g^	1.07 ± 0.00^a^	1.16 ± 0.03^b^	1.50 ± 0.01^b^	23.77 ± 0.01^e^
Average	1.84 ± 0.01	1.83 ± 0.01	1.85 ± 0.02	0.08 ± 0.01	0.05 ± 0.01	0.03 ± 0.00	0.53 ± 0.02	0.04 ± 0.01	0.05 ± 0.01	1.24 ± 0.01	1.22 ± 0.01	1.45 ± 0.01	1.17 ± 0.01	1.88 ± 0.03	1.36 ± 0.01	6.18 ± 1.70

Values are presented as mean value of three replicates followed by ± standard error. Different letters in a column indicate significant variation (*p* ≤ 0.05) according to Duncan's multiple‐range test (DMRT).

The quantification of anthraquinones (alizarin, purpurin, and mollugin) is of particular importance as these are the important pharmacological constituents of *R. cordifolia*. Significantly (*p* ≤ 0.05) higher content of alizarin in leaves (1.73 mg/g dw) and roots (2.68 mg/g dw) was recorded in the RC‐9 population, while in the stems, the highest alizarin content (1.68 mg/g dw) was reported in RC‐5 and RC‐9 populations. Purpurin content in the leaves (1.30 mg/g dw), and roots (3.68 mg/g dw) was significantly (*p* ≤ 0.05) higher in the RC‐3 population, while, in the stems, higher purpurin content (1.43 mg/g dw) was recorded in the RC‐6 population. Mollugin was detected only in the roots, and significantly (*p* ≤ 0.05) higher content (23.77 mg/g dw) was recorded in the RC‐9 population. However, mollugin was not detected in RC‐2, RC‐3, RC‐6, and RC‐7 populations. Factorial analysis also revealed that these bioactive compounds showed significant (*p* ≤ 0.05) interactions between plant parts and populations (Table S1).

### 2.6 Antioxidant Activity

The antioxidant capacity of the extracts, assessed using ABTS, DPPH, and FRAP assays, generally followed trends similar to those observed for phenolic and flavonoid contents and also varied significantly (*p* ≤ 0.05) among populations (Table 6). ABTS scavenging activity was highest in leaves of the RC‐5 population (9.04 mmol AAE/g), whereas the RC‐9 population contained the greatest activity in stems (6.64 mmol AAE/g) and roots (18.24 mmol AAE/g), with root activity notably exceeding that of the RC‐1 population (13.29 mmol AAE/g). Although the RC‐9 population showed the strongest DPPH scavenging activity in leaves (2.01 mmol AAE/g). Stems exhibited peak activity in the RC‐2 population (1.52 mmol AAE/g), while roots displayed comparatively uniform activity, with RC‐2, RC‐6, and RC‐8 populations showing similar higher values (∼1.27 mmol AAE/g). FRAP values were consistently highest in the RC‐9 population across leaves (1.06 mmol AAE/g), stems (0.87 mmol AAE/g), and roots (1.31 mmol AAE/g), indicating higher reducing capacity in this population.

**TABLE 6:** Antioxidant activity in the different populations of *R. cordifolia*.

Population Code	ABTS (mmol AAE/g)			DPPH (mmol AAE/g)			FRAP (mmol AAE/g)		
	Leaf	Stem	Root	Leaf	Stem	Root	Leaf	Stem	Root
RC‐1	8.54 ± 0.10 ^de^	4.89 ± 0.08^c^	13.29 ± 0.07^b^	1.71 ±0.00^c^	1.21 ±0.00^d^	0.90 ±0.01^a^	0.43 ±0.00^c^	0.24 ±0.00^b^	0.79 ±0.00^g^
RC‐2	8.44 ± 0.07^d^	4.15 ± 0.07^ab^	14.34 ± 0.09^c^	1.96 ±0.00^g^	1.52 ±0.00^i^	1.27 ±0.02^f^	0.59 ±0.00^f^	0.24 ±0.00^b^	0.72 ±0.00^f^
RC‐3	8.93 ± 0.03^fg^	4.32 ± 0.07^b^	15.75 ± 0.10^e^	1.84 ± 0.01^f^	1.29 ± 0.01^f^	1.25 ± 0.00^f^	0.39 ± 0.00^b^	0.21 ± 0.00^a^	0.51 ± 0.00^c^
RC‐4	7.64 ± 0.04^b^	3.84 ± 0.03^a^	12.75 ± 0.04^a^	1.52 ± 0.01^a^	0.94 ± 0.00^a^	1.12 ± 0.00^d^	0.50 ± 0.00^e^	0.24 ± 0.00^b^	0.33 ± 0.00^a^
RC‐5	9.04 ± 0.03^g^	4.84 ± 0.04^c^	14.78 ± 0.06^d^	1.81 ± 0.01^e^	1.31 ± 0.01^g^	1.08 ± 0.00^c^	0.39 ± 0.00^b^	0.24 ± 0.00^b^	0.67 ± 0.00^d^
RC‐6	8.19 ± 0.01^c^	6.01 ± 0.07^d^	15.57 ± 0.01^e^	1.78 ± 0.00^d^	1.25 ± 0.00^e^	1.27 ± 0.00^f^	0.47 ± 0.00^d^	0.24 ±0.00^b^	0.71 ± 0.00^e^
RC‐7	6.54 ± 0.11^a^	6.01 ± 0.10^d^	13.50 ± 0.02^b^	1.62 ± 0.01^b^	1.07 ± 0.01^b^	1.06 ± 0.00^b^	0.29 ± 0.00^a^	0.24 ± 0.00^b^	0.45 ± 0.00^b^
RC‐8	8.06 ± 0.09^c^	4.98 ± 0.07^c^	17.95 ± 0.13^f^	1.71 ± 0.01^d^	1.16 ± 0.01^c^	1.27 ± 0.01^f^	1.00 ± 0.00^g^	0.81 ± 0.00^c^	1.05 ± 0.00^h^
RC‐9	8.71 ± 0.11^ef^	6.64 ± 0.39^d^	18.24 ± 0.03^g^	2.01 ± 0.01^h^	1.49 ± 0.02^h^	1.22 ± 0.02^e^	1.06 ± 0.01^h^	0.87 ± 0.02^d^	1.31 ± 0.01^i^
Average	8.23 ± 0.14	5.04 ± 0.17	15.15 ± 0.36	1.76 ± 0.03	1.25 ± 0.03	1.16 ± 0.02	0.57 ± 0.05	0.37 ± 0.00	0.73 ± 0.05

Values are presented as mean value of three replicates followed by ± standard error. Different letters in a column indicate significant variation (*p* ≤ 0.05) according to Duncan's multiple‐range test (DMRT).

### 2.7 Habitat‐Dependent Variation

Habitat type significantly influenced soil properties, nutritional composition, active phytochemicals, and antioxidant activity of the *R. cordifolia*. Moist shady habitats represented the highest soil moisture (16.51%), organic carbon (4.94%), total nitrogen (0.48%), and potassium (0.29%), whereas shady habitats represented the highest pH (6.89), and open grassy habitats contained the greatest phosphorus content (0.09%) (Table S2). Nutritional constituents were consistently higher under plant growing in moist shady conditions, with highest total carbohydrates (leaf: 2.27 mg/g; stem: 2.27 mg/g; root: 0.29 mg/g dw), proteins (leaf: 0.204 mg/g; stem: 0.103 mg/g; root: 0.090 mg/g dw), free amino acids (leaf: 1.96 mg/g; stem: 1.75 mg/g; root: 3.09 mg/g dw), ascorbic acid (leaf: 5.65 mg/g; stem: 5.76 mg/g; root: 4.75 mg/g dw), and thiamine (leaf: 1.71 mg/g; stem: 1.45 mg/g; root: 1.80 mg/g dw) (Figure 1A). Presence of antinutritional factors also varied by habitat conditions of plants. Phytic acid peaked in leaves under shady habitat (1.31 mg/g dw), stems of open grassy habitat (1.11 mg/g dw), and roots of plant in moist shady condition (1.72 mg/g dw), while trypsin inhibitory activity was highest in leaves (2.91 mg/g dw) and stems (1.93 mg/g dw) of plant growing in open grassy habitats, but was highest in roots of plant growing under moist shady conditions (1.04 mg/g dw) (Figure 1B).

**FIGURE 1:**
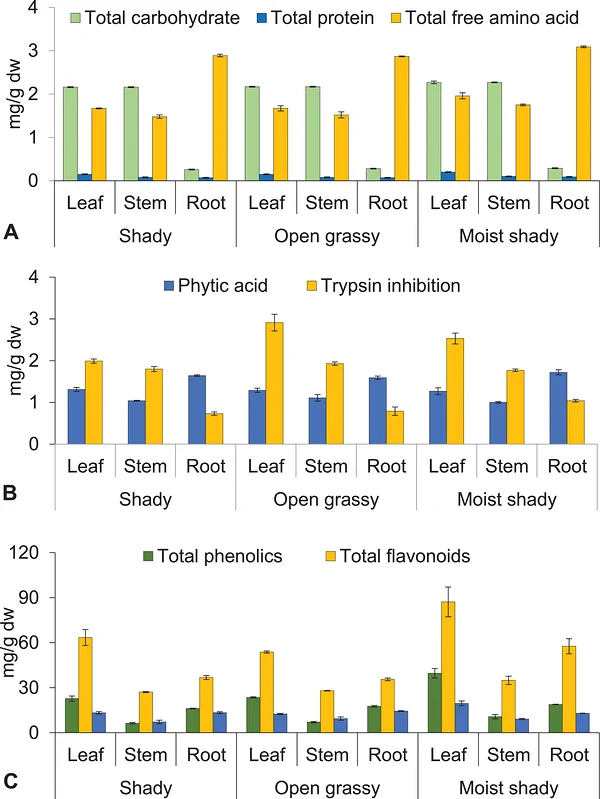
Nutritional profile (A) and antinutritional profile (B) and phenolics (total phenolics, flavonoids, and tannins) of (C) *R. cordifolia* among different habitat conditions.

Metabolite concentrations were strongly habitat‐dependent. Moist shady populations showed the highest accumulation of total phenolics (leaf: 39.65 mg GAE/g; stem: 10.69 mg GAE/g; root: 18.83 mg GAE/g dw), flavonoids (leaf: 87.15 mg QE/g; stem: 34.88 mg QE/g; root: 57.59 mg QE/g dw), and leaf tannins (19.59 mg TAE/g), whereas stem (9.41 mg TAE/g dw) and root tannins (14.44 mg TAE/g dw) were highest in open grassy habitats (Figure 1C).

Polyphenolic and major active compounds measured by HPLC also varied markedly among habitats (Table S3). For example, gallic acid peaked in plants growing in moist shady habitats (leaf: 1.88 mg/g; stem: 1.89 mg/g; root: 1.97 mg/g dw). Catechin content was highest in leaves of moist shady grown plants (0.09 mg/g), while similar levels were observed in stems from plants of open grassy and moist shady habitats (0.06 mg/g dw), and were equal in roots from shady and moist shady habitats (0.03 mg/g dw). Salicylic acid was highest in leaves of plants grown in shady habitats (0.72 mg/g dw), but in stems (0.21 mg/g dw) and roots (0.08 mg/g dw) it peaked in moist shady habitats. Alizarin was highest in leaves (1.58 mg/g) and stems (1.45 mg/g dw) of plants in moist shady habitats, while alizarin peaked in roots of plantgrown in shady habitats (1.69 mg/g dw). Purpurin reached highest values in leaves of plants of shady habitats (1.20 mg/g dw), stems in moist shady habitats (1.25 mg/g dw), and roots in open grassy habitats (2.73 mg/g dw). Mollugin, detected only in roots, was highest in plants growing in shady habitats.

Antioxidant activity demonstrated clear habitat‐specific patterns (Figure 2). ABTS activity was highest in leaves of plants growing in open grassy habitats (8.8 mmol AAE/g dw), whereas stems (5.77 mmol AAE/g dw) and roots (17.25 mmol AAE/g dw) exhibited highest activity in plants of moist shady habitats. DPPH activity peaked in open grassy leaves (1.87 mmol AAE/g dw) and stems (1.37 mmol AAE/g dw), while roots showed the highest activity in plants of moist shady habitats (1.25 mmol AAE/g dw). FRAP activity was consistently highest in plants of moist shady populations across all tissues (leaf: 0.85 mmol AAE/g; stem: 0.64 mmol AAE/g; root: 1.02 mmol AAE/g dw), indicating higher reducing capacity under shade conditions.

**FIGURE 2:**
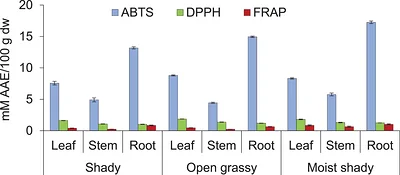
Antioxidant activities using ABTS, DPPH, and FRAP assays in *R. cordifolia* among different habitat conditions.

### 2.8 Relationship of Edaphic Factors With Nutritional, Chemical Constituents, and Antioxidant Activity

Pearson correlation analysis showed that altitude was significantly associated (*p* ≤ 0.05) with most biochemical and soil parameters across all plant parts. In leaves, altitude was positively correlated with accumulation of total phenolics (*p* ≤ 0.01; *r* = 0.812), flavonoids (*p* ≤ 0.01; *r* = 0.820), tannins (*p* ≤ 0.01; *r *= 0.885), alizarin (*p* ≤ 0.01; *r* = 0.847), catechin (*p* ≤ 0.05; *r* = 0.467) and antioxidant activity (FRAP: *p* ≤ 0.01; *r* = 0.585). Nutritional traits such as total carbohydrate (*p* ≤ 0.01; *r* = 0.604), protein (*p* ≤ 0.01; *r* = 0.935), free amino acid (*p* ≤ 0.01; *r* = 0.937), thiamine (*p* ≤ 0.01; *r *= 0.541), as well as soil moisture (*p* ≤ 0.01; *r* = 0.693), organic carbon (*p* ≤ 0.05; *r* = 0.481), and nitrogen (*p* ≤ 0.05; *r* = 0.453), also showed positive correlation with altitude. Conversely, salicylic acid (*p* ≤ 0.05; *r* = −0.618) and soil pH (*p* ≤ 0.05; *r* = −0.680) correlated negatively with altitude (Figure 3A).

**FIGURE 3:**
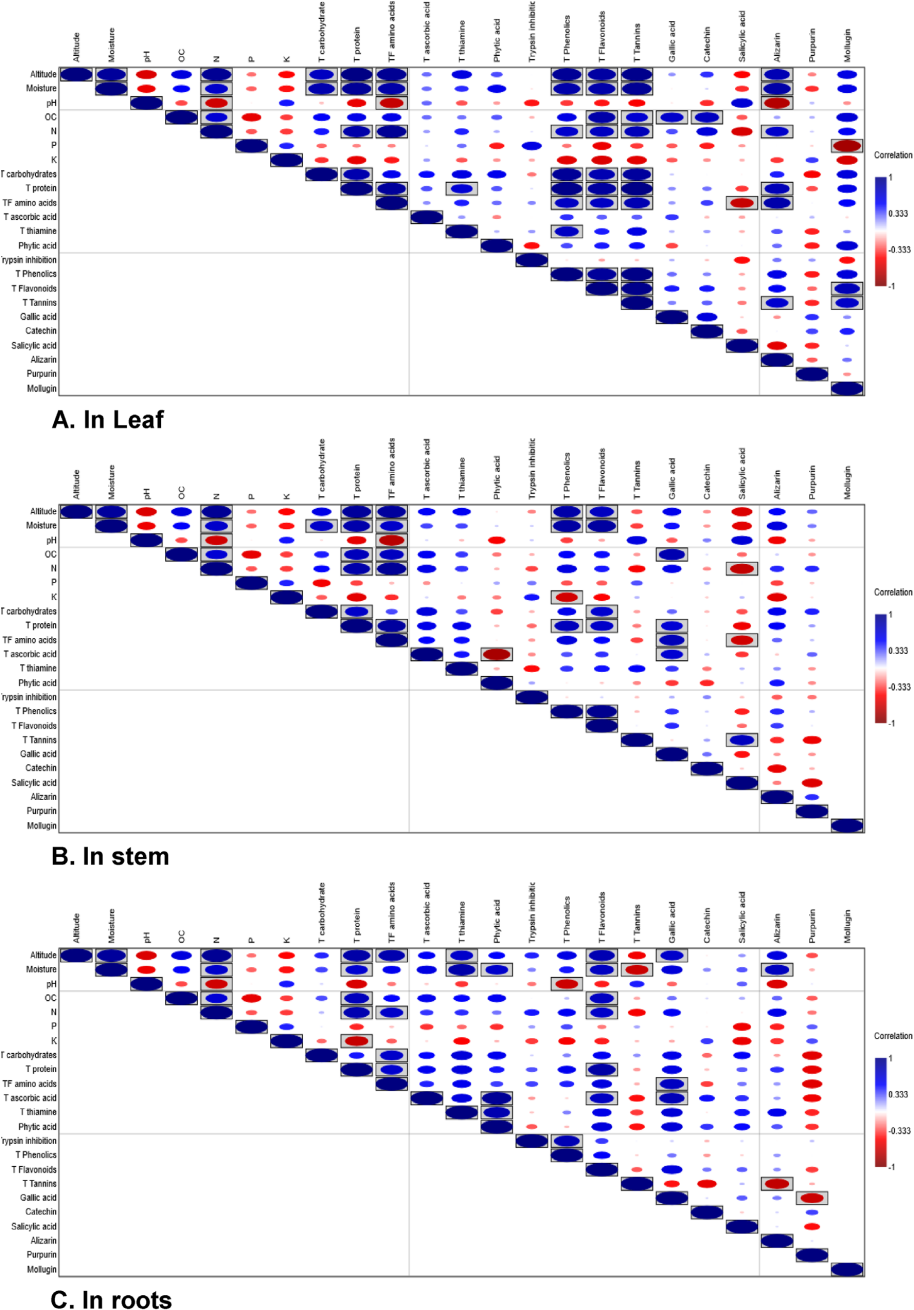
Correlation among altitude, polyphenolics, antioxidant activity, nutritional, antinutritional profile and edaphic factors in *Rubia cordifolia* leaves (a); correlation among altitude, polyphenolics, antioxidant activity, nutritional, antinutritional profile and edaphic factors in *R. cordifolia* stems (b); correlation among altitude, polyphenolics, antioxidant activity, nutritional, antinutritional profile and edaphic factors in *R. cordifolia* roots (c); abbreviation used: N‐Total nitrogen; N‐Total phosphorus; P‐Total potassium; OC‐Organic carbon; T Phenolics—Total phenolics; T flavonoids‐Total flavonoids; T tannins ‐Total tannins; T carbohydrate ‐Total carbohydrate; T protein‐Total protein; TF amino acid‐Total free amino acid; T ascorbic acid ‐Total ascorbic acid; T thiamine ‐Total thiamine; Trypsin inhibition‐Trypsin inhibitory activity.

In stems, altitude was positively associated with phenolics (*p* ≤ 0.01; *r* = 0.733), flavonoids (*p* ≤ 0.01; *r* = 0.625) gallic acid (*p* ≤ 0.01; *r* = 0.600), alizarin (*p* ≤ 0.01; *r* = 0.518), ABTS (*p* ≤ 0.01; *r* = 0.685), FRAP (*p* ≤ 0.01; *r* = 0.732), carbohydrates (*p* ≤ 0.01; *r* = 0.604), proteins (*p* ≤ 0.01; *r* = 0.928), free amino acids (*p* ≤ 0.01; *r* = 0.927), ascorbic acid (*p* ≤ 0.01; *r* = 0.498), soil moisture (*p* ≤ 0.01; *r* = 0.693), and moderate positive correlations with thiamine (*p* ≤ 0.05; *r* = 0.482), organic carbon (*p* ≤ 0.05; *r *= 0.481), and nitrogen (*p* ≤ 0.05; *r *= 0.453). Negative correlations occurred with tannins (*p* ≤ 0.05; *r* =−0.447), salicylic acid (*p* ≤ 0.01; *r* = −0.729) and soil pH (*p* ≤ 0.01; *r* = −0.680) (Figures 3B).

In roots, altitude was positively correlated with phenolics (*p* ≤ 0.01; *r* = 0.621), flavonoids (*p* ≤ 0.01; *r* = 0.797), gallic acid (*p* ≤ 0.01; *r* = 0.694), alizarin (*p* ≤ 0.01; *r* = 0.646), mollugin (*p* ≤ 0.01; *r* = 0.523), ABTS (*p* ≤ 0.01; *r* = 0.674), FRAP (*p* ≤ 0.01; *r* = 0.502), protein (*p* ≤ 0.01; *r* = 0.812), thiamine (*p* ≤ 0.01; *r* = 0.594), soil moisture (*p* ≤ 0.01; *r* = 0.653), and free amino acids (*p* ≤ 0.01; *r* = 0.699). Moderate positive correlation was observed with ascorbic acid (*p* ≤ 0.05; *r* = 0.465), phytic acid (*p* ≤ 0.05; *r* = 0.415), trypsin inhibitors (*p* ≤ 0.05; r = 0.398), organic carbon (*p* ≤ 0.05; *r *= 0.481), and nitrogen (*p* ≤ 0.05; *r* = 0.453). In contrast, negative correlations appeared with tannins (*p* ≤ 0.01; *r* = −0.612), and soil pH (*p* ≤ 0.05; *r* = −0.680) (Figure 3C).

Edaphic factors also demonstrated strong associations with nutrients and phytochemicals. Soil moisture displayed positive correlations with phenolics, flavonoids, alizarin, antioxidant activities (ABTS & FRAP) across plant parts, and major nutritional components (carbohydrates, proteins, and free amino acids), while showing negative correlations with salicylic acid (stem) and tannins (root). Soil organic carbon positively correlated with plant flavonoids, tannins, gallic acid, catechin, proteins, free amino acids, and ascorbic acid. Soil pH generally exhibited negative correlations with phenolics, flavonoids, alizarin, proteins, and moisture, but positive correlations with salicylic acid and tannins. Total phosphorus showed positive correlations with ABTS and trypsin inhibitors in leaves, and negative correlations with moisture, catechin, and mollugin in other plant parts. Soil total nitrogen consistently correlated positively with protein, free amino acids, soil moisture, and organic carbon across plant parts. Total potassium displayed positive correlations with FRAP, ascorbic acid, gallic acid, flavonoids, and DPPH, and negative correlations with ABTS, phytic acid, and tannins (Figure 3A–C).

## 3 Discussion

The pronounced interpopulation variability observed in the chemical composition and biological activities of *R. cordifolia* collected from diverse altitudinal ranges (1300–2500 m asl) and habitat conditions (moist shady, shady, and open grassy) was strongly association with altitude and the physicochemical properties of the soil. In this study, clear elevation‐linked gradients were evident in soil parameters, with higher altitude sites exhibiting increased moisture content, organic carbon, and nitrogen, patterns consistent with prior Himalayan research [21, 22]. The presence of tree species like Banj–Oak and Himalayan cypress at upper elevations may enhance water retention and litter input [23], while lower temperatures at these altitudes may reduce microbial decomposition, enabling organic matter accumulation and higher carbon stocks [24]. Oak‐dominated forests at mid‐ to high‐altitudes contribute nutrient‐rich litter that improves soil fertility relative to pine‐dominated lower elevations [25]. These soil conditions were associated with enhanced accumulation of carbohydrates, proteins, and free amino acids in high‐altitude populations, suggesting that edaphic factors and elevation‐driven fertility influence primary metabolic output. Concurrently, decreasing soil pH with altitude, potentially associated with higher rainfall, dense canopy, and organic matter buildup [26], was negatively correlated with most metabolites, indicating that slightly acidic soils favor the biosynthesis of phenolics, flavonoids, and anthraquinones. Acidic conditions improve micronutrient availability and activate phenylpropanoid pathway enzymes [25], thereby enhancing secondary metabolite production.

Edaphic characteristics emerged as strong predictors of metabolite accumulation. Significantly positive relationships between soil moisture and bioactive metabolites, antioxidant capacity, and nutritional constituents are consistent with earlier studies showing that water availability enhances metabolite production in tea (*Camellia sinensis*) and other medicinal herbs [26]. The positive correlation of organic carbon content with flavonoids, tannins, proteins, and amino acids has also been reported in *Rhodiola sachalinensis* and *Taxus wallichiana* with organic carbon serving as a proxy for soil fertility [27]. Conversely, the negative correlation of soil pH with phytochemical and nutritional traits may be consistent with the known suppression of nutrient uptake under alkaline conditions and the promotive role of acidic soils in metabolite biosynthesis [4]. Phosphorus and potassium exhibited compound‐specific relationships, possibly reflecting their regulatory roles in energy metabolism, signal transduction, and enzyme activity. For instance, phosphorus negatively correlated with catechin and mollugin in roots, aligning with findings in *Arabidopsis thaliana* where phosphorus depletion modulates metabolite accumulation [28]. Soil nitrogen positively influenced leaf protein and amino acid levels, consistent with studies in *Taxus wallichiana* and *Rhodiola* spp. [27, 29]. Collectively, these results indicate that edaphic conditions, including moisture, pH, carbon, and nutrients, are associated with climatic variables to regulate metabolic allocation.

The results support the fundamental principle that medicinal plants show significant metabolic plasticity in response to environmental fluctuations. This aligns with extensive evidence indicating that both primary and secondary plant metabolites are shaped by ecological pressures, resource availability, and environmental conditions essential for plant survival under heterogeneous climatic regimes [30, 31]. Secondary metabolites demonstrated higher sensitivity to environmental variables than primary nutrients. Total phenolics, flavonoids, tannins, and specific bioactive compounds varied significantly across altitude and habitat types, reinforcing the hypothesis that secondary metabolism is tightly regulated by abiotic stress gradients. In our findings, altitude showed strong positive correlations with phenolics, flavonoids, alizarin, catechin, and mollugin across all plant parts, suggesting that high‐altitude stress induces protective metabolite accumulation. High altitudes, with elevated UV‐B exposure, temperature fluctuations, and oxidative stress, are known to upregulate phenylpropanoid pathways [32]. This pattern is well‐documented in other Himalayan species, including *Hedychium spicatum, Valeriana jatamansi* [32, 33]. Antioxidant capacity closely mirrored polyphenolic content in which ABTS and FRAP activity correlated strongly with altitude. The enhanced biosynthesis of protective metabolites (phenolics, flavonoids, and anthraquinones), nutritional composition, and antioxidant activity in these environments may reflect evolutionary adaptations that increase resilience under adverse conditions. Although certain compounds (e.g., salicylic acid in leaves and stems, total tannins in stems) decreased with altitude, this may indicate trade‐offs or feedback responses under extreme conditions.

The strong positive correlations between antioxidant activity and phenolic content are mechanistically supported by the redox properties of polyphenols, which act as hydrogen donors, electron donors, and metal chelators [34]. The structure–activity relationship of phenolics, governed by hydroxyl group positioning, glycosylation, and other substituents, further explains variability in antioxidant strength [35]. These molecules mitigate oxidative stress through multiple mechanisms, including free radical scavenging, enzyme inhibition, metal ion binding, and upregulation of endogenous antioxidant enzymes [36]. Their role in human health is well established, with daily intake recommendations ranging from 25 to 1000 mg for phenolics and 50 to 800 mg for flavonoids [37]. In addition to elevation, microhabitat conditions were associated with phytochemical patterns. Moist shady habitats consistently supported higher levels of total phenolics, flavonoids, gallic acid, and alizarin. These habitats offer moderated temperature, elevated humidity, and rich organic matter, sustaining metabolic activity and reducing oxidative stress. Enhanced FRAP activity paralleled the increased accumulation of phenolics and flavonoids in moist shady populations. ABTS activity peaked in open grassy leaves, while moist shady stems and roots also showed high values, revealing tissue‐specific pattern of antioxidant strategies. DPPH activity, more influenced by light‐exposed tissues, was highest in open grassy populations. Such conditions also enhanced nutrient uptake, explaining higher levels of carbohydrates, proteins, free amino acids, ascorbic acid, and thiamine in plants from these habitats.

These findings align with ecological theories that predict greater allocation to primary metabolism under benign conditions [38]. In contrast, open grassy habitats, characterized by greater irradiance and lower moisture, were associated with increased tannin concentrations in stems and roots. This is consistent with light‐induced activation of tannin biosynthesis as a defense against UV radiation and herbivory [39]. Notably, antinutritional factors such as phytic acid and trypsin inhibitors also showed distinct habitat‐linked patterns: trypsin inhibitor activity peaked in open grassy conditions (possibly in response to herbivory), whereas phytic acid was more abundant in moist shady roots. Importantly, the overall nutritional content remained higher than antinutritional factors across all habitats, supporting the dietary and medicinal relevance of *R. cordifolia*. Plants at high altitudes face multiple abiotic stressors, including temperature extremes, UV‐B radiation, hypoxia, and nutrient limitations, all of which influence gene expression, signaling networks, and metabolic regulation [1, 40]. Importantly, phytochemical variation does not manifest uniformly across plant organs. Leaves, stems, and roots respond differently to environmental signals depending on their functional roles and exposure levels. Hence, whole‐plant analyses remain essential for understanding environmental responses in medicinal plants.

## 4 Conclusion

The present study concluded that environmental factors, including altitude, habitat type, and edaphic conditions, significantly influenced the metabolic plasticity of *R. cordifolia* across natural populations of the Western Himalaya. The findings highlighted that environmental heterogeneity was associated with the accumulation of both nutritional constituents and secondary metabolites, leading to significant variations in metabolite composition and antioxidant activity among populations. The study further revealed significant relationships between environmental variables and metabolite accumulation. Higher‐altitude populations growing under moist shady habitats with greater soil moisture, organic carbon, and nitrogen exhibited higher contents of phenolics, flavonoids, anthraquinones (alizarin, purpurin, and mollugin), and antioxidant activity, whereas soil pH showed largely negative associations with many metabolites. These results highlighted the association of demonstrate the important role of environmental conditions in determining the medicinal quality of *R. cordifolia*. Based on the key findings of the present work, high‐altitude populations and moist shady habitats can be considered promising sources of germplasm for conservation, cultivation, and sustainable utilization. The study provides an ecological and phytochemical knowledge base for selecting elite populations with superior medicinal potential and establishes a basis for conservation strategies, and quality standardization of *R. cordifolia*.

## 5 Materials and Methods

### 5.1 Literature Review

To understand the current status of information on this species, a literature search was conducted to explore various aspects of R. cordifolia in four databases (Google Scholar, Web of Science, Scopus, and Pubmed). In addition, cross‐references of relevant literature were thoroughly examined. A total of 119 research articles were selected for this study covering diverse areas such as traditional/ethnomedicinal uses (37 articles), nutritional and phytochemical contents (22 articles), and pharmacological activities (60 articles).

### 5.2 Study Sites, Plant Material, and Sampling Design

Whole plants of *R. cordifolia* were collected from nine natural locations during August 2018 distributed across diverse altitudinal ranges (1300–2500 m asl) and habitat conditions (moist shady, shady, and open grassy) in the Kumaon region of Uttarakhand, Western Himalaya. The populations were designated as RC‐1 to RC‐9 (RC‐1: Kosi, RC‐2: Dwarahat, RC‐3: Semalkote, RC‐4: Lobanj, RC‐5: Berinag, RC‐6: Nag, RC‐7: Tallital‐Nainital, RC‐8: Mallital‐Nainital, RC‐9: Dunagiri) and arranged according to increasing altitude. Details of study sites including altitude, latitude, longitude, habitat, and forest types are presented in Table 1.

The study followed a factorial sampling design involving two factors: population (nine natural populations) and plant part (leaf, stem, and root). At each population, 15–20 healthy individuals were surveyed, and plants were randomly selected, maintaining a minimum distance of 20–25 m between sampled individuals to ensure independent sampling and minimize the likelihood of collecting closely associated or clonal plants. Three independent biological replicates were prepared for each population from randomly selected individuals and transported to the laboratory for analysis. The study therefore consisted of 09 populations × 03 plant parts (leaf, stem, and root) × 03 biological replicates, resulting in 81 observations for each analyzed parameter. The plant parts (leaves, stems, and roots) from each biological replicate were separated, thoroughly washed with tap water followed by distilled water, and dried at room temperature (25 ± 4°C; relative humidity 40 ± 5%) for 10 days until a constant weight was achieved. The dried samples were ground into a fine powder using a mechanical grinder and stored in airtight containers at 4°C until further analysis to maintain sample integrity.

Simultaneously, soil samples were collected from the rhizosphere of each biological replicate using a soil core sampler. The samples were transported to the laboratory in airtight polyethylene bags and air‐dried at room temperature for 1 week at room temperature, gently ground using a wooden roller and pestle, and freed from stones, roots, leaves, and other debris. The processed soil was passed through a 0.2‐mm sieve, packed in airtight zip‐lock bags, and stored at room temperature until physicochemical analysis.

### 5.3 Chemicals and Reagents

2,4,6‐tripyridyl‐s‐triazine (TPTZ), 2,2‐azino‐bis‐3‐ethylbenzthiazoline 6‐sulphonic acid (ABTS), 2,2‐diphenyl‐1‐picryhydrazyl (DPPH) radicals and standards such as gallic acid, quercetin, tannic acid, catechin, salicylic acid, alizarin, purpurin, and mollugin were procured from Sigma‐Aldrich (St. Louis, Missouri, United States). The solvents viz. ethanol, acetone, and methanol were procured from Hi‐Media Laboratories Pvt. Ltd. (Mumbai, India). All the other chemicals and solvents were of analytical grade.

### 5.4 Edaphic Factors

To understand the influence of soil chemistry on plant metabolism, physicochemical parameters of the soil from each collection site were estimated. Soil moisture, pH, organic carbon (OC), total nitrogen (N), total potassium (K), and total phosphorus (P) were determined using standard pedological methods as described by Nath et al. [41].

### 5.5 Nutritional and Antinutritional Content

Standard biochemical methodologies were employed to measure total carbohydrate, total protein, free amino acid, ascorbic acid (Vitamin C), and thiamine (Vitamin B1) content. To assess nutrient bioavailability and potential dietary limitations, antinutritional factors such as phytic acid and trypsin inhibitory activity were estimated following the protocols outlined by Jugran et al. [42].

### 5.6 Extraction for Phytochemicals and Antioxidant Activity

Prior to the analysis, the finely ground powder samples (0.25 g) were extracted with 35 mL of 70 % ethanol (v/v). Each sample extract was heated at 60°C for 60 min in a water bath and allowed to stand on the rotary shaker for 24 h (160 rpm) at room temperature (∼25°C). The temperature and duration chosen for the study (60°C for 60 min followed by shaking for 24 h at room temperature) have been frequently used for extraction of bioactive compounds as they offer improved solvent penetration and solubility with minimal degradation of heat‐sensitive compounds [43]. The homogenized mixture was centrifuged at room temperature for 20 min at 10000 rpm. The supernatant was filtered and stored at 4°C prior to phytochemical analysis

### 5.7 Determination of Total Phenolic, Flavonoid, and Tannin Contents

Spectrophotometric assays were used for the gross quantification of major phytochemical classes, including total phenolic content (TPC), estimated using the Folin–Ciocalteu method and expressed as gallic acid equivalents (GAE); total flavonoid content (TFC), determined using the aluminum chloride colorimetric method and expressed as quercetin equivalents (QE); and total tannin content (TTC), measured using the Folin–Denis reagent and expressed as tannic acid equivalents (TAE) [35].

### 5.8 HPLC‐DAD Analysis of Bioactive Compounds

Quantification of phenolic compounds (gallic acid, catechin, and salicylic acid) and the active compounds of *R. cordifolia* (alizarin, purpurin, and mollugin) was performed using high‐performance liquid chromatography (HPLC, Alliance Waters e2695, Waters Milford, USA) equipped with a photodiode detector (PDA, Waters 2998). An isocratic elution system was used to separate individual compounds on Waters SPHERISORB C18(5 µm particle size, 4.6 × 250 mm i.d.) at 30°C. The injection volume was 10 µL. The mobile phase consisted of methanol (Merck HPLC grade) and water (40:60 v/v), acidified with 0.1% o‐phosphoric acid to suppress the ionization of phenolic acids. The total run time of 50 min was used to isolate the compounds at 0.8 mL/min. Calibration curves were constructed using different concentrations of standard compounds (gallic acid, catechin, and salicylic acid) ranging from 10 to 50 ppm. The standard equations obtained from the calibration curves were used for quantification of the above compounds in different samples. Results were expressed as mg/g dw of *R. cordifolia* (leaves, stems, and roots) extracts [44]. Quantification of alizarin, purpurin, and mollugin was carried out following the procedure of Zarkogianni et al. [45] with minor modification. Isocratic elution was performed using a mixture of acetonitrile (ACN) and water containing 0.1% trifluoroacetic acid (TFA) in a ratio of 55:45 v/v. The flow rate was 1.5 mL/min and the analysis was completed within a run time of 35 min. Specific wavelengths were selected for optimal detection: 247 nm for alizarin and mollugin, and 255 nm for purpurin. The compounds were identified based on their retention times: 4.2 min for alizarin, 5.5 min for purpurin, and 27.4 min for mollugin.

### 5.9 Antioxidant Activity

The antioxidant potential of the extracts was evaluated using three complementary in vitro assays to capture different mechanisms of radical scavenging and reducing power. The assays included the 2,2′‐azino‐bis (3‐ethylbenzothiazoline‐6‐sulfonic acid) (ABTS) radical scavenging assay, the 2,2‐diphenyl‐1‐picrylhydrazyl (DPPH) radical scavenging assay, and the ferric reducing antioxidant power (FRAP) assay [46]. Results were presented in millimoles (mM) ascorbic acid equivalents per g dry weight.

### 5.10 Statistical Analysis

Data for soil physicochemical properties, nutritional composition, phytochemical constituents, bioactive compounds, and antioxidant activities were expressed as mean ± standard error (SE) of three independent biological replicates. The study followed a factorial sampling design with two fixed factors: population (nine natural populations) and plant part (leaf, stem, and root). The effects of population, plant part, and their interaction (Population × Plant Part) were evaluated using two‐way factorial analysis of variance (ANOVA) in IBM SPSS Statistics (*version 26*, IBM Corp., Armonk, NY, USA). Whenever significant differences were detected (*p* < 0.05), mean separation was performed using Duncan's Multiple‐Range Test (DMRT). Pearson's correlation coefficients among edaphic factors, nutritional traits, phytochemical constituents, and antioxidant activities were calculated using IBM SPSS Statistics (*version 26*). Correlation matrices were visualized as heatmaps using PAST (*version 6.0*, Paleontological Statistics Software Package for Education and Data Analysis), whereas other graphical illustrations were prepared using Microsoft Excel 2010.

## Author Contributions

**Deepti Tiwari**: conceptualization, data curation, formal analysis, methodology, writing – original draft. **Sandeep Rawat**: conceptualization, methodology, data curation, investigation. **Veena Pandey**: methodology, data curation, formal analysis. **Vinod C. Joshi**: data curation, formal analysis. **Indra D. Bhatt**: conceptualization, investigation, funding acquisition, project administration, resources, supervision, writing – review and editing. **R.C. Sundriyal**: funding acquisition, investigation, writing – review & editing.

## Conflicts of Interest

The authors declare no conflicts of interest.

## Ethical Statements

No animal or human subjects were involved in the present study.

## Supporting information




**Supporting File 1**: cbdv71747‐sup‐0001‐SuppMat.docx

## Data Availability

The data that support the findings of this study are available from the corresponding author upon reasonable request.

## References

[1] D. Tiwari , P. Kewlani , K. S. Gaira , I. D. Bhatt , R. C. Sundriyal , and V. Pande , “Predicting Phytochemical Diversity of Medicinal and Aromatic Plants (MAPs) across Eco‐climatic Zones and Elevation in Uttarakhand Using Generalized Additive Model,” Scientific Reports 13 (2023): 10888, 10.1038/s41598-023-37495-1. PMC10322824 37407604

[2] P. Pant , S. Pandey , and S. DallAcqua , “The Influence of Environmental Conditions on Secondary Metabolites in Medicinal Plants: A Literature Review,” Chemistry and Biodiversity 18 (2021): e2100345, 10.1002/cbdv.202100345. 34533273

[3] A. K. Jugran , A. Bahukhandi , P. Dhyani , I. D. Bhatt , R. S. Rawal , and S. K. Nandi , “Impact of Altitudes and Habitats on Valerenic Acid, Total Phenolics, Flavonoids, Tannins, and Antioxidant Activity of *Valeriana jatamansi* ,” Applied Biochemistry and Biotechnology 179 (2016): 911–926, 10.1007/s12010-016-2039-2. 26971960

[4] A. Setyawati , Komariah , B. Pujiasmanto , A. Fatawi , and I. Batubara , “Secondary Metabolites of Turmeric and Ginger on Various Altitudes and Soil Characteristics,” in IOP Conference Series: Earth and Environmental Science (2021), 1–15, 10.1088/1755-1315/724/1/012020.

[5] H. C. Andola , K. S. Gaira , R. S. Rawal , M. S. M. Rawat , and I. D. Bhatt , “Habitat‐dependent Variations in berberine Content of *Berberis Asiatica* Roxb. *Ex*. DC. in Kumaon, Western Himalaya,” Chemistry and Biodiversity 7 (2010): 415–420, 10.1002/cbdv.200900041. 20151388

[6] S. Rawat , A. K. Jugran , I. D. Bhatt , R. S. Rawal , and S. K. Nandi , “Effects of Genetic Diversity and Population Structure on Phenolic Compounds Accumulation in Hedychium Spicatum,” Ecological Genetics and Genomics 3 (2017): 25–33, 10.1016/j.egg.2017.06.003.

[7] K. Hazra , D. Kumar , A. K. Meena , P. Sharma , and G. Babu , “Application of Thin Layer Chromatography in Determining the Natural Diversifications of Phytochemicals in *Rubia Cordifolia* ,” Current Spectroscopy and Chromatography 11 (2025): E29504910378059.

[8] N. Deshkar , S. Tilloo , and V. Pande , “A Comprehensive Review of *Rubia Cordifolia* Linn,” Pharmacognosy Reviews 2 (2008): 124, 10.2174/0129504910378059250714100323.

[9] M. Wen , Q. Chen , W. Chen , et al., “A Comprehensive Review of *Rubia Cordifolia* L.: Traditional Uses, Phytochemistry, Pharmacological Activities, and Clinical Applications,” Frontiers in Pharmacology 13 (2022): 965390, 10.3389/fphar.2022.965390. PMC9500525 36160419

[10] A. Ali , M. Aslam , and S. S. Chaudhary , “A Review on Pharmacognostic and Therapeutic Uses of *Rubia Cordifolia* ,” Journal of Drug Delivery and Therapeutics 10 (2020): 195–202, 10.22270/jddt.v10i6.4514.

[11] S. Mishra , S. Sonter , M. K. Dwivedi , and P. K. Singh , “Anti‐sickling, Anti‐inflammatory and Anti‐oxidant Potential of *Rubia Cordifolia* L.: A Traditionally Used Medicinal Plant of Amarkantak, Madhya Pradesh (India),” Medicinal Plants 13 (2021): 499–514, 10.5958/0975-6892.2021.00058.7.

[12] D. Sharma , D. Jain , and D. Bhatia , “Unveiling the Modern Therapeutic Properties and Folk Medicinal Riches of *Rubia Cordifolia* ,” Recent Patents on Biotechnology 20 (2025): 242–254, 10.2174/0118722083359338250611055647. 41992461

[13] R. Patil , M. Mohan , V. Kasture , and S. Kasture , “ *Rubia Cordifolia*: A Review,” Advances in Traditional Medicine 9 (2009): 1–13, 10.3742/OPEM.2009.9.1.001.

[14] K. Khan , R. Karodi , A. Siddiqui , S. Thube , and R. Rub , “Development of Anti‐acne Gel Formulation of Anthraquinones Rich Fraction From *Rubia Cordifolia* (Rubiaceae),” International Journal of Applied Research in Natural Products 4 (2011): 28–36.

[15] A. Siddiqui , K. M. Y. Amin , R. H. Zuberi , and A. Jamal , “ *Standardization of Majith* (*Rubia cordifolia* Linn.),” Indian Journal of Traditional Knowledge 10 (2011): 330–333.

[16] K. Mohanraj , B. S. Karthikeyan , R. P. Vivek‐Ananth , et al., “IMPPAT: A Curated Database of Indian Medicinal Plants, Phytochemistry and Therapeutics,” Scientific Reports 8 (2018): 4329, 10.1038/s41598-018-22631-z. PMC5847565 29531263

[17] B. S. Chandrashekar , S. Prabhakara , T. Mohan , et al., “Characterization of *Rubia Cordifolia* L. Root Extract and Its Evaluation of Cardioprotective Effect in Wistar Rat Model,” Indian Journal of Pharmacology 50 (2018): 12–21, 10.4103/ijp.IJP_418_17. PMC5954628 29861523

[18] P. Kaur , M. Chandel , S. Kumar , N. Kumar , B. Singh , and S. Kaur , “Modulatory Role of Alizarin From Rubia cordifolia L. Against Genotoxicity of Mutagens,” Food and Chemical Toxicology 48 (2010): 320–325, 10.1016/j.fct.2009.10.019. 19852997

[19] M. N. Watroly , M. Sekar , S. Fuloria , et al., “Chemistry, Biosynthesis, Physicochemical and Biological Properties of Rubiadin: A Promising Natural Anthraquinone for New Drug Discovery and Development,” Drug Design, Development and Therapy 15 (2021): 4527–4549, 10.2147/DDDT.S338548. PMC8576757 34764636

[20] A. Verma , B. Kumar , P. Alam , V. Singh , and S. K. Gupta , “ *Rubia Cordifolia*‐a Review on Pharmacognosy and Phytochemistry,” International Journal of Pharmaceutical Sciences and Research 7 (2016): 2720.

[21] A. Kumar , M. Kumar , R. Pandey , Y. ZhiGuo , and M. Cabral‐Pinto , “Forest Soil Nutrient Stocks along Altitudinal Range of Uttarakhand Himalaya: An Aid to Nature Based Climate Solutions,” Catena 207 (2021): 105667, 10.1016/j.catena.2021.105667.

[22] P. Kewlani , V. S. Negi , I. D. Bhatt , R. S. Rawal , and S. K. Nandi , “Soil Nutrients Concentration Along Altitudinal Gradients in Indian Western Himalaya,” Scandinavian Journal of Forest Research 36 (2021): 98–104, 10.1080/02827581.2020.1871065.

[23] C. M. Sharma , S. Gairola , S. K. Ghildiyal , and S. Suyal , “Physical Properties of Soils in Relation to Forest Composition in Moist Temperate Valley Slopes of the Central Western Himalaya,” Journal of Forest and Environmental Science 26 (2010): 117–129.

[24] S. Zhang , D. Chen , D. Sun , X. Wang , J. L. Smith , and G. Du , “Impacts of Altitude and Position on the Rates of Soil Nitrogen Mineralization and Nitrification in Alpine Meadows on the Eastern Qinghai–Tibetan Plateau, China,” Biology and Fertility of Soils 48 (2012): 393–400, 10.1007/s00374-011-0634-5.

[25] M. A. Sheikh and M. Kumar , “Nutrient Status and Economic Analysis of Soils in oak and Pine Forests in Garhwal Himalaya,” Journal of American Science 6 (2010): 117–121.

[26] E. K. Cheruiyot , L. M. Mumera , W. K. Ng'etich , A. Hassanali , and F. Wachira , “Polyphenols as Potential Indicators for Drought Tolerance in Tea (*Camellia sinensis* L.),” Bioscience, Biotechnology, and Biochemistry 71 (2007): 2190–2197, 10.1271/bbb.70156. 17827703

[27] P. Adhikari , K. Joshi , M. Singh , and A. Pandey , “Influence of Altitude on Secondary Metabolites, Antioxidants, and Antimicrobial Activities of Himalayan Yew (*Taxus wallichiana*),” Plant Biosystems 156 (2022): 187–195, 10.1080/11263504.2020.1845845.

[28] G. Hernandez‐Ramirez , S. M. Brouder , D. R. Smith , and G. E. Van Scoyoc , “Greenhouse Gas Fluxes in an Eastern Corn Belt Soil: Weather, Nitrogen Source, and Rotation,” Journal of Environmental Quality 38 (2009): 841–854, 10.2134/jeq2007.0565. 19329673

[29] X. Yan , S. Wu , Y. Wang , X. Shang , and S. Dai , “Soil Nutrient Factors Related to Salidroside Production of *Rhodiola Sachalinensis* Distributed in Chang Bai Mountain,” Environmental and Experimental Botany 52 (2004): 267–276, 10.1016/j.envexpbot.2004.02.005.

[30] A. Szakiel , C. Pączkowski , and M. Henry , “Influence of Environmental Abiotic Factors on the Content of Saponins in Plants,” Phytochemistry Reviews 10 (2011): 471–491, 10.1007/s11101-010-9177-x.

[31] Y. Li , D. Kong , Y. Fu , M. R. Sussman , and H. Wu , “The Effect of Developmental and Environmental Factors on Secondary Metabolites in Medicinal Plants,” Plant Physiology and Biochemistry 148 (2020): 80–89, 10.1016/j.plaphy.2020. 31951944

[32] C. Cirak , J. Radusiene , V. Jakstas , L. Ivanauskas , F. Seyis , and F. Yayla , “Altitudinal Changes in Secondary Metabolite Contents of *Hypericum Androsaemum* and *Hypericum Polyphyllum* ,” Biochemical Systematics and Ecology 70 (2017): 108–115, 10.1016/j.bse.2016.11.006.

[33] R. Suyal , S. Rawat , R. S. Rawal , and I. D. Bhatt , “Variability in Morphology, Phytochemicals, and Antioxidants in *Polygonatum Verticillatum* (L.) All. Populations under Different Altitudes and Habitat Conditions in Western Himalaya, India,” Environmental Monitoring and Assessment 191 (2019): 1–18, 10.1007/s10661-019-7687-6. 31989296

[34] B. Kaurinovic and D. Vastag , Flavonoids and Phenolic Acids as Potential Natural Antioxidants (IntechOpen, 2019), 1–20, 10.5772/intechopen.83731.

[35] S. Rawat , P. Acharya , P. O. Bhutia , et al., “Changes in Nutritional, Physicochemical, Phytochemical Composition and Antioxidant Potential of *Mahonia Nepalensis* Fruits during Ripening,” International Journal of Food Properties 26 (2023): 1062–1078, 10.1080/10942912.2023.2200480.

[36] D. Krishnaiah , R. Nithyanandam , and R. Sarbatly , “Phytochemical Constituents and Activities of *Morinda Citrifolia* L. ,” in Phytochemicals‐a Global Perspective of Their Role in Nutrition and Health, ed. A. V. Rao (Intechopen Publishers, 2012), 127–150, 10.5772/26094.

[37] C. D. Stalikas , “Extraction, Separation, and Detection Methods for Phenolic Acids and Flavonoids,” Journal of Separation Science 30 (2007): 3268–3295, 10.1002/jssc.200700261. 18069740

[38] L. Hua , J. Gao , M. Zhou , and S. Bai , “ *Impacts of Relative Elevation on Soil Nutrients and Apple Quality in the Hilly‐gully Region of the Loess Plateau*, China,” Sustainability 13 (2021): 1293, 10.3390/su13031293.

[39] A. R. War , M. G. Paulraj , T. Ahmad , et al., “Mechanisms of Plant Defense against Insect Herbivores,” Plant Signaling and Behavior 7 (2012): 1306–1320, 10.4161/psb.21663. PMC3493419 22895106

[40] R. Kumar and M. Kumari , “Adaptive Mechanisms of Medicinal Plants along Altitude Gradient: Contribution of Proteomics,” Biologia Plantarum 62 (2018): 630–640, 10.1007/s10535-018-0817-0.

[41] S. Nath , S. Rawat , R. S. Rawal , I. D. Bhatt , B. Pathak , and M. H. Fulekar , “Soil Constituents Influence Accumulation of Phytochemicals and Nutritional Content in *Wrightia Tinctoria* of North Gujarat, India,” Indian Journal of Plant Physiology 22 (2017): 197–205, 10.1007/s40502-017-0297-9.

[42] A. K. Jugran , W. Y. Chaudhary , A. Bahukhandi , I. D. Bhatt , R. S. Rawal , and P. P. Dhyani , “Effect of Processing and Storage Methods on the Nutritional, Anti‐nutritional, and Anti‐oxidant Properties of *Paeonia emodi*, Wall. Ex. Royle,” Applied Biochemistry and Biotechnology 180 (2016): 322–337, 10.1007/s12010-016-2101-0. 27146874

[43] G. A. Akowuah and I. Zhari , “Effect of Extraction Temperature on Stability of Major Polyphenols and Antioxidant Activity of Orthosiphon Stamineus Leaf,” Journal of Herbs, Spices, & Medicinal Plants 16 (2010): 160–166, 10.1080/10496475.2010.509652.

[44] T. Belwal , P. Dhyani , I. D. Bhatt , R. S. Rawal , and V. Pande , “Optimization Extraction Conditions for Improving Phenolic Content and Antioxidant Activity in Berberis asiatica Fruits Using Response Surface Methodology (RSM),” Food Chemistry 207 (2016): 115–124, 10.1016/j.foodchem.2016.03.081. 27080887

[45] M. Zarkogianni , Z. E. Papliaka , and E. Tsatsaroni , “Identification and Quantitative Determination of Madder by High Performance Liquid Chromatography: Application to Historical Textiles,” Journal of Liquid Chromatography and Related Technologies 32 (2009): 2334–2345, 10.1080/10826070903187874.

[46] I. D. Bhatt , S. Rawat , A. Badhani , and R. S. Rawal , “Nutraceutical Potential of Selected Wild Edible Fruits of the Indian Himalayan Region,” Food Chemistry 215 (2017): 84–91, 10.1016/j.foodchem.2016.07.143. 27542453

