# Supplementary material for: Environmental Drivers of Metabolic Plasticity in Rubia cordifolia From the Western Himalaya: Implications for Phytochemical Diversity and Antioxidant Potential
Source: Chem Biodivers. 2026 Sep 30;23(10):e71747. doi: 10.1002/cbdv.71747 (PMC13627966; doi:10.1002/cbdv.71747)
Supplement: Supplementary file 1 [file CBDV-23-e71747-s001.docx]

**Supplementary Materials**

**Table S1:** Polyphenols, major active compounds, nutritional, anti-nutritional profile, and antioxidant activity in different plant parts of *R. cordifolia*

| **Parameters** | **Leaf** | **Stem** | **Root** |
| --- | --- | --- | --- |
| **Polyphenolics:** |  |  |  |
| Total phenol (mg GAE/g) | 28.63 ±1.92 | 8.07 ±0.63 | 17.53 ±0.05 |
| Total flavonoids (mg QE/g) | 68.11 ±4.53 | 29.99 ±1.12 | 43.31 ±2.60 |
| Total tannins (mg TAE/g) | 15.11 ±0.87 | 8.56 ±0.56 | 13.59 ±0.25 |
| Gallic acid (mg/g) | 1.84 ±0.03 | 1.83 ±0.01 | 1.85 ±0.02 |
| Catechin (mg/g) | 0.08 ±0.01 | 0.05 ±0.00 | 0.03 ±0.00 |
| Salicylic acid (mg/g) | 0.53 ±0.09 | 0.04 ±0.01 | 0.05 ±0.01 |
| **Major active compounds:** |  |  |  |
| Alizarin (mg/g) | 1.24 ±0.07 | 1.22 ±0.08 | 1.45 ±0.18 |
| Purpurin (mg/g) | 1.17 ±0.01 | 1.88 ±0.04 | 1.36 ±0.00 |
| Mollugin (mg/g) | ND | ND | 6.18 ±1.70 |
| **Nutritive and anti-nutritional constituents:** |  |  |  |
| Total carbohydrate (mg/g) | 2.20 ±0.01 | 1.07 ±0.00 | 0.28 ±0.20 |
| Total protein (mg/g) | 0.172 ±0.00 | 0.092 ±0.00 | 0.077 ±0.00 |
| Total free amino acid (mg/g) | 1.77 ±0.04 | 1.58 ±0.03 | 2.95 ±0.02 |
| Total ascorbic acid (mg/g) | 5.59 ±0.02 | 5.61 ±0.03 | 4.68 ±0.01 |
| Total thiamine (mg/g) | 1.65 ±0.01 | 1.38 ±0.01 | 1.78 ±0.01 |
| Phytic acid (mg/g) | 1.29 ±0.03 | 1.05 ±0.02 | 1.65 ±0.02 |
| Trypsin inhibitory activity (mg/g) | 2.48 ±0.10 | 1.83 ±0.03 | 0.85 ±0.04 |
| **Antioxidant activity:** |  |  |  |
| ABTS activity (mM AAE/g) | 8.23 ±0.14 | 5.04 ±0.17 | 15.15 ±0.36 |
| DPPH activity (mM AAE/g) | 1.76 ±0.03 | 1.25 ±0.03 | 1.16 ±0.02 |
| FRAP activity (mM AAE/g) | 0.57 ±0.05 | 0.37 ±0.00 | 0.73 ±0.05 |

Values are reported as the mean of three replicates ± standard error; ND - Not detected

**Table S2.** Physicochemical characteristics of soil under different habitat conditions of *R. cordifolia*

| **Habitat conditions** | **Moisture**  **(%)** | **pH** | **Organic carbon (%)** | **Total nitrogen (%)** | **Total phosphorus (%)** | **Total potassium (%)** |
| --- | --- | --- | --- | --- | --- | --- |
| Shady | 15.21 ±0.31 | 6.89 ±0.17 | 4.65 ±0.24 | 0.41 ±0.04 | 0.05 ±0.00 | 0.28 ±0.01 |
| Open-grassy | 15.07 ±0.29 | 6.75 ±0.15 | 4.49 ±0.25 | 0.36 ±0.04 | 0.09 ±0.01 | 0.27 ±0.01 |
| Moist-shady | 16.51 ±0.51 | 6.56 ±0.08 | 4.94 ±0.24 | 0.48 ±0.06 | 0.08 ±0.01 | 0.30 ±0.01 |

Values are presented as mean ± standard error of three replicates.

**Table S3.** Polyphenols and bioactive compounds in *R. cordifolia* among different habitat conditions

| Polyphenols  & bioactive  compounds | Shady | | | Open grassy | | | Moist shady | | |
| --- | --- | --- | --- | --- | --- | --- | --- | --- | --- |
|  | Leaf | Stem | root | Leaf | Stem | Root | Leaf | Stem | Root |
| Gallic acid | 1.82 ±0.5 | 1.77 ±0.3 | 1.81 ±0.01 | 1.81 ±0.06 | 1.84 ±0.2 | 1.75 ±0.03 | 1.88 ±0.06 | 1.89 ±0.2 | 1.97 ±0.01 |
| Catechin | 0.04 ±0.04 | 0.04 ±0.00 | 0.03 ±0.00 | 0.05 ±0.01 | 0.06 ±0.00 | 0.02 ±0.01 | 0.09 ±0.01 | 0.06 ±0.00 | 0.03 ±0.01 |
| Salicylic acid | 0.72 ±0.23 | 0.07 ±0.03 | 0.07 ±0.03 | 0.54 ±0.10 | 0.06 ±0.03 | ND | 0.32 ±0.10 | 0.21 ±0.02 | 0.08 ±0.04 |
| Alizarin | 1.08 ±0.10 | 1.37 ±0.02 | 1.69 ±0.34 | 1.05 ±0.16 | 0.95 ±0.18 | 1.10 ±0.34 | 1.58 ±0.03 | 1.45 ±0.10 | 1.59 ±0.27 |
| Purpurin | 1.20 ±0.01 | 1.24 ±0.02 | 2.01 ±0.26 | 1.17 ±0.03 | 1.01 ±0.08 | 2.73 ±0.29 | 1.14 ±0.21 | 1.25 ±0.06 | 1.68 ±0.06 |
| Mollugin | ND | ND | 9.71 ±2.60 | ND | ND | 0.91 ±0.45 | ND | ND | 7.94 ±3.97 |

Values are presented as mean value of three replicates followed by ± standard error. ND- Not Detected
